# Methotrexate enhances the anti-inflammatory effect of CF101 via up-regulation of the A_3 _adenosine receptor expression

**DOI:** 10.1186/ar2078

**Published:** 2006-11-13

**Authors:** Avivit Ochaion, Sara Bar-Yehuda, Shira Cohn, Luis Del Valle, Georginia Perez-Liz, Lea Madi, Faina Barer, Motti Farbstein, Sari Fishman-Furman, Tatiana Reitblat, Alexander Reitblat, Howard Amital, Yair Levi, Yair Molad, Reuven Mader, Moshe Tishler, Pnina Langevitz, Alexander Zabutti, Pnina Fishman

**Affiliations:** 1 Can-Fite Biopharma Ltd., 10 Bareket Street, Kiryat-Matalon, Petah-Tikva, 49170, Israel; 2 The Mina and Everard Goodman Faculty of Life Sciences, Bar-Ilan University, Heren Hyesod Street, Ramat-Gan, 5200, Israel; 3 Department of Neuroscience, Neuropathology Core & Center for NeuroVirology, Temple University School of Medicine, North 12th Street, Philadelphia, PA 19122, USA; 4 Internal Department D, The Barzilai Medical Center, Hahistadrut Street, Ashkelon, 78278, Israel; 5 Internal Department D/E, Meir Medical Center, Tshernihovsky Street, Kfar Saba, 44281, Israel; 6 Rheumatology Department, Rabin Medica Center, Zabutinsky Street, Petah-Tikva, 49100, Israel; 7 Medical Clinic of Rheumatology, Ha'Emek Medical Center, Afula, 18101 Israel; 8 Rheumatology Department, Assaf Harofeh Medical Center, Zerifin, Beer Yakov, 70300, Israel; 9 Internal Department F, The Chaim Sheba Medical Center, Tel-Hashomer, 52621 Israel

## Abstract

Methotrexate (MTX) exerts an anti-inflammatory effect via its metabolite adenosine, which activates adenosine receptors. The A_3 _adenosine receptor (A_3_AR) was found to be highly expressed in inflammatory tissues and peripheral blood mononuclear cells (PBMCs) of rats with adjuvant-induced arthritis (AIA). CF101 (IB-MECA), an A_3_AR agonist, was previously found to inhibit the clinical and pathological manifestations of AIA. The aim of the present study was to examine the effect of MTX on A_3_AR expression level and the efficacy of combined treatment with CF101 and MTX in AIA rats. AIA rats were treated with MTX, CF101, or both agents combined. A_3_AR mRNA, protein expression and exhibition were tested in paw and PBMC extracts from AIA rats utilizing immunohistochemistry staining, RT-PCR and Western blot analysis. A_3_AR level was tested in PBMC extracts from patients chronically treated with MTX and healthy individuals. The effect of CF101, MTX and combined treatment on A_3_AR expression level was also tested in PHA-stimulated PBMCs from healthy individuals and from MTX-treated patients with rheumatoid arthritis (RA). Combined treatment with CF101 and MTX resulted in an additive anti-inflammatory effect in AIA rats. MTX induced A_2A_AR and A_3_AR over-expression in paw cells from treated animals. Moreover, increased A_3_AR expression level was detected in PBMCs from MTX-treated RA patients compared with cells from healthy individuals. MTX also increased the protein expression level of PHA-stimulated PBMCs from healthy individuals. The increase in A_3_AR level was counteracted *in vitro *by adenosine deaminase and mimicked *in vivo *by dipyridamole, demonstrating that receptor over-expression was mediated by adenosine. In conclusion, the data presented here indicate that MTX induces increased A_3_AR expression and exhibition, thereby potentiating the inhibitory effect of CF101 and supporting combined use of these drugs to treat RA.

## Introduction

Low-dose methotrexate (MTX) is the most widely used antirheumatic drug and it is the 'gold standard' against which other systemic medications are compared [1]. It has as its target the enzyme dihydrofolate reductase, which is required for reduction of dihydrofolate to tetrahydrolate. It is presumed that cells exposed to MTX die as a result of reduced folate depletion [2]. Adenosine, an additional active metabolite of MTX, has been found to have potent anti-inflammatory effects, and earlier studies [3,4] strongly support the notion that the anti-inflammatory effect of MTX is attributed more to adenosine than to tetrahydrolate. MTX increases the extracellular concentration of adenosine, where it is known to exert its anti-inflammatory effect via suppression of inflammatory cytokines such as tumour necrosis factor (TNF)-α, interleukin-6, or macrophage inhibitory protein-1α [5-7]. It was further found that the anti-inflammatory effect of adenosine is mediated via A_2A _and the A_3 _adenosine receptors [8,9].

The highly selective A_3 _adenosine receptor (A_3_AR) agonist IB-MECA (1-deoxy-1-[6-[[(3-iodophenyl)methyl]amino]-9H-9-yl]-*N*-methyl-β-D-ribofura-nuronamide) had an anti-inflammatory effect in collagen-induced arthritis in DBA1 mice and adjuvant-induced arthritis (AIA) in rats [10,11]. Interestingly, A_3_AR was found to be over-expressed in the synovial and paw inflammatory tissues as compared with corresponding tissues in normal, healthy animals. Moreover, receptor upregulation was also identified in the peripheral blood mononuclear cells (PBMCs) of AIA rats compared with control animals. Mechanistically, on treatment with IB-MECA, downregulation of A_3_AR expression level was noted in cells derived from the synovial tissue, most probably due to receptor internalization and degradation. Subsequently, decreased levels of expression of phosphatidylinositol-3 kinase and protein kinase B/Akt were observed. The latter of these proteins is known to control the nuclear factor-κB (NF-κB) signal transduction pathway. The decreased levels of PKB/Akt resulted in failure to phosphorylate IKK, which in turn resulted in inability to release NF-κB from its IκB complex. These events led to decreased expression of NF-κB and TNF-α, resulting in apoptosis of synovial cells. Remarkably, the PBMCs of AIA rats responded to IB-MECA treatment in the same manner as did the synovial cells, namely with receptor downregulation, suggesting that PBMCs reflect the receptor situation in inflammatory tissues and may have utility as a biomarker for monitoring response to IB-MECA [12]. Furthermore, Gessi and coworkers [13] recently noted upregulation of A_3_AR expression in phytohemagglutinin (PHA)-stimulated PBMCs from healthy individuals. It thus seems that A_3_AR expression correlates with cell activation or pathogenicity.

Recently, IB-MECA (commercially known as CF101) was tested in phase I clinical trials in healthy individuals. CF101, in single and multiple oral dose studies, was found to be safe and well tolerated, and the pharmacokinetics were linearly proportional to dose [14]. In an early phase II clinical trial of CF101 conducted in patients with rheumatoid arthritis (RA), the drug was well tolerated and conferred benefit as monotherapy [15].

Most biological disease-modifying antirheumatic drugs currently are given in combination with MTX [16]. Therefore, the aim of the present study was to evaluate the efficacy of combined MTX+CF101 treatment. We found that MTX treatment increased expression of A_3_AR, rendering inflammatory cells more susceptible to CF101. Combined treatment of AIA rats with MTX and CF101 enhanced the anti-inflammatory effect of each drug. In addition, we found A_3_AR to be over-expressed in PBMCs of MTX-treated patients and in activated cells from healthy individuals. The molecular mechanisms involved are explored.

## Materials and methods

### Reagents

The A_3_AR agonist IB-MECA was synthesized for Can-Fite BioPharma Ltd (Petah-Tikva, Israel) by Albany Molecular Research Inc (Albany, NY, USA) and is referred to as CF101 in the following text. A stock solution of 10 mmol/l was prepared in DMSO and further dilutions were performed. RPMI, foetal bovine serum and antibiotics for cell cultures were obtained from Beit Haemek (Haifa, Israel). The A_3_AR antagonist MRS1523 was purchased from Sigma (St. Louis, MO, USA) and diluted in the same manner as for CF101. Rabbit polyclonal antibodies against rat and human A_3_AR as well as rat A_2A_AR were purchased from Santa Cruz Biotechnology Inc. (Santa Cruz, CA, USA). MTX, PHA, adenosine and adenosine deaminase (ADA) were purchased from ABIC (Beit Shemesh, Israel). Dipyridamole was purchased from Sigma.

### Effect of CF101 and MTX on development of AIA

Female Lewis rats, aged 9 weeks, were obtained from Harlan Laboratories (Jerusalem, Israel). Rats were maintained on a standardized pellet diet and supplied with tap water. Experiments were performed in accordance with the guidelines established by the Institutional Animal Care and Use Committee at Can-Fite BioPharma, Petach Tikva, Israel. The rats were injected subcutaneously at the tail base with 100 μl suspension composed of incomplete Freund's adjuvant with 10 mg/ml heat killed *Mycobacterium tuberculosis *H37Ra (Difco, Detroit, MI, USA). Each group included 10 animals.

For the prophylactic treatment study, MTX treatment (0.25 mg/kg intraperitoneally) was administered once weekly, starting 3 days after disease induction. CF101 (100 μg/kg) was orally administered by gavage, twice daily, starting at disease onset. The study also included a group treated with a combination of MTX and CF101. The control group received vehicle only (DMSO at a dilution corresponding to that of CF101).

For the therapeutic treatment study, MTX (0.75 mg/kg intraperitoneally, once weekly) as well as CF101 (100 μg/kg orally, twice daily) were initiated on disease onset. As in the prophylactic study, a group treated with a combination of MTX and CF101 was included.

Clinical disease activity score was assessed as follows. The animals were inspected every day for clinical arthritis. The scoring system ranged from 0 to 4 of each limb: 0 indicates no arthritis, 1 indicates redness or swelling of one toe/finger joint, 2 indicates redness and swelling of more than one toe/finger joints, 3 indicates involvement of ankle and tarsal-metatarsal joints, and 4 indicates redness or swelling of the entire paw. The four individual leg scores were added together to yield a total clinical score.

At the end of the study the legs were removed up to the knees, fixed in 10% formaldehyde, decalcified, dehydrated, paraffin embedded and cut into 4 μm sections. They were then stained by haematoxylin and eosin, and morphopathological assessment was performed.

Pathological assessments were performed using semiquantitative grading scales from 0 to 4 for the following parameters: the extent of inflammatory cell infiltration into the joint tissues, synovial lining cell hyperplasia, pannus formation, joint cartilage layer destruction and bone damage and erosion: 0 indicates normal; 1 indicates minimal loss of cortical bone at a few sites; 2 indicates mild loss of cortical trabecular bone; 3 indicates moderate loss of bone at many sites; 4 indicates marked loss of bone at many sites; and 5 indicates marked loss of bone at many sites, with fragmenting and full thickness penetration of inflammatory process or pannus into the cortical bone. The means of all of the histological parameter scores were summated to yield an overall 'histology score'.

In addition, blood samples were collected and subjected to Ficoll hypaque gradient. The PBMCs were then washed with phosphate-buffered saline (PBS), and protein extracts were prepared as detailed below.

The hind paws were dissected above the ankle joint. The bony tissue was broken into pieces, snap frozen in liquid nitrogen and stored at -80°C until use. The paw tissues were added to RIPA extraction buffer (4 ml/g tissue) contaning 150 mmol/l NaCl, 50 mmol/l Tris, 1% NP40, 0.5% deoxycholate and 0.1% SDS. Tissues were homogenized on ice with a polytron, centrifuged and the supernatants were subjected to Western Blot analysis.

### Effect of dipyridamole on the expression of A_3_AR in PBMCs from naïve rats

Dipyridamole (5 mg/kg intraperitoneally) was administered once to naïve rats and blood samples were drawn 2, 6, 24 and 48 hours after dipyridamole administration and subjected to Ficoll hypaque gradient. The PBMCs were then washed with PBS and protein extracts were prepared as detailed below.

### Immunohistochemistry staining of paraffin-embedded slides of paws tissues derived from AIA rats

The paraffin on the slides was melted from the sections, which were placed in xylene three times for 30 min each. The tissues were hydrated with serial dilutions of ethanol and then antigen was retrieved by heating with citrate buffer at 95°C for 30 min. The slides were allowed to cool down and then washed three times in PBS. Endogenous peroxidase quenching was performed by washing the sections with fresh 20% hydrogen peroxide in methanol for 20 min. The sections were then blocked by incubating in 5% normal goat serum in PBS-BSA 0.1% for 2 hours.

The primary antibody (Novus Biologicals Inc., Littleton, CO, USA) was diluted in 0.1% PBS-BSA and incubated overnight at room temperature. After three washes in 1× PBS, the slides were incubated in 0.5% biotinilated secondary antibody in PBS-BSA 0.1% for 1 hour at room temperature and then subjected to an avidin-biotin complex After another wash the slides were incubated with DAB substrate, washed in tap water (to dispose of the DAB) and inactivated with bleach. A light haematoxylin counterstain was performed and the haematoxylin was then removed by quick dip in acid alcohol and the slides were washed in ammonia water, dehydrated and mounted with Permount.

### Human blood sample collection and separation

Blood samples were collected from healthy individuals and from patients with RA who were either newly and chronically treated with MTX. Protocols for the study were approved by the hospitals' ethical committees as was the blood sample collection. Healthy individuals and RA patients provided signed, informed consent prior to blood withdrawal. Patient characteristics are summarized in Table 1.

To separate PBMCs, heparanized blood (20 ml) was subjected to Ficoll hypaque gradient. The PBMCs were then washed with PBS and subjected to the various assays.

### Double immunofluorecence staining of PBMCs of RA patients

PBMCs (1 × 10^6 ^cells) were smeared on electrophorized glass slide and fixed with 75% ethanol for 2 min. The slides were then blocked with normal horse serum for 1 hour at room temperature in a humidified chamber, after which a mouse monoclonal CD4 (Santa Cruz Biotechnology; Clone MT310, 1:50 dilution) primary antibody was incubated overnight at room temperature. After rinsing thoroughly with PBS, a rhodamine-tagged secondary antibody (Vector Laboratories, Burlingame, CA, USA; 1:200 dilution) was incubated overnight in the dark. Then the slides were rinsed with PBS, blocked with normal goat serum and incubated with a rabbit polyclonal anti-A_3_AR antibody (Chemicon International, Temecula, CA, USA; AB9111, 1:100 dilution) overnight. Cells were rinsed with PBS and a second, fluorescein-tagged anti-rabbit antibody was incubated for 1 hour in the dark. Finally, slides were rinsed, coverslipped with an aquous based mounting media (Vectashield; Vector Laboratories) and visualized in a Nikon ultraviolet inverted microscope and processed with a deconvolution software (Slidebook 4.0; Intelligent Imaging, Denver, CO, USA).

### Activation of PBMCs

PBMCs (2 × 10^6 ^cells/ml) from healthy individuals or from MTX-treated RA patients were incubated in cell cultures with RPMI 1640 supplemented with 10% FBS. PBMCs from healthy individuals were activated with PHA (5 μg/ml) for 24 hours. MTX (1 μmol/l) was added for an additional 27 hours. ADA (1 unit/ml), CF101 (10 nmol/l), or MRS1353 (10 nmol/l) was added to the culture system for the last 3 hours. In another set of experiments PBMCs from healthy individuals were incubated for 27 hours with adenosine (25 μmol/l), and ADA (1 unit/ml) was added to the culture system for the last 3 hours. PBMCs from MTX-treated RA patients were incubated for 1 hour with CF101 (10 nmol/l). At the end of the incubation time in all of the above experiments, the PBMCs were collected from the culture plates and protein extracts were prepared.

### Western blot analysis of A_3_AR and additional signalling proteins in PBMCs

Western blot analyses were carried out according to the following protocol. Samples were rinsed with ice-cold PBS and transferred to ice-cold lysis buffer (TNN buffer: 50 mmol/l Tris buffer [pH 7.5], 150 mmol/l NaCl and NP40). Cell debris was removed by centrifugation for 10 min at 7500 *g*. Protein concentrations were determined using the BioRad protein assay dye reagent (BioRad Laboratories, Hercules, CA, USA). Equal amounts of the sample (50 μg) were separated by SDS-PAGE, using 12% polyacrylamide gels. The resolved proteins were then electroblotted onto nitrocellulose membranes (Schleicher & Schuell, Keene, NH, USA). Membranes were blocked with 1% BSA and incubated with the desired primary antibody (dilution 1:1000) for 24 hours at 4°C. Blots were then washed and incubated with a secondary antibody for 1 hour at room temperature. Bands were recorded using BCIP/NBT color development kit (Promega, Madison, WI, USA).

Analysis of A_3_AR protein expression level in patient PBMCs was performed as follows. Samples from each of four RA patients were run in the same gel with a pool of samples from four healthy individuals, designated the standard. The blots were quantified by densitometric analysis and the ratio of RA patient/standard was calculated. Blots of mitogen stimulated cells were quantified against β-actin. The data presented in the figures are representative of at least three different experiments.

### RT-PCR analysis of formalin-fixed paraffin-embedded paw tissue slides

Tissue sections of paws derived from AIA rats treated with vehicle, MTX, CF101, or MTX+CF101 were mounted on slides and then deparaffinized in xylen and rehydrated by washing in serial dilutions of ethanol. Slides were used immediately or stored at -80°C until use. After rehydration, 20 μl of solution A (1.25× PCR buffer [200 mmol/l Tris-HCl, 500 mmol/l KCl], 6.25 mmol/l MgCl_2_, 5 U RNasin [Promega], 2 mmol/l DTT, 1 U RQ1 RNase-free DNase [Promega]) was directly applied to the marked area. The marked area was completely scraped off the slide using a pipette tip, and neoplastic tissue or normal tissue was collected into different microcentrifuge tubes. The samples were treated with proteinase K at a final concentration of 0.1 mg/ml. The samples were incubated at 37°C for 1 hour to allow for DNA digestion. Cells lysate were heated to 95°C for 15 min in order to inactivate DNase and proteinase K. Following centrifugation at 14,000 rpm for 5 min, 17 μl of the supernatant was transferred to a separate tube and 4 μl of RT mixure (5 mmol/l dNTPs, 2.5 μmol/l random hexamer, 5 U RNasin, 100 U SuperScript One Step RT-PCR with Platinum Taq (Invitrogene, San Diago, CA, USA) and the primers for rat A_3_AR (245 up CTA GCA CTG GCA GAC and 245 down CAG CAG AGG CCC AGG) were added.

The RT reaction was performed at 45°C for 45 min, followed by heating to 99°C for 5 min; then, 50 cycles at 94°C for 30 s, 50°C for 75 s and 73°C for 45 s, and an extension of 73°C for 7 min were performed. Products were electrophoresed on 2% agarose gels, stained with ethidium bromide and visualized with ultraviolet illumination. The specificity of the RT-PCR reaction was confirmed by size determination on agarose gels in comparison with a positive control, from RNA extracted using standard techniques, and by sequencing the RT-PCR product and comparing the sequences with the known sequences (ADORA3-L77729, L77730). The optical density of the bands (Et-Br) was quantified using an image analysis system. This analysis was performed on tissues from three different experiments.

### Statistical analysis

To analyze differences in clinical score between the four study groups (CF101, MTX, MTX+CF101 [combined treatment] and control), we used analysis of variance (ANOVA) with repeated measures from days 8 to 25 for prophylactic treatment and from days 14 to 20 for therapeutic treatment. To test differences in trends during the study between the four study groups, we used ANOVA using Dunnet method to evaluate differences between each of the study groups and the control group: for prophylactic treatment this was from days 20 to 25, adjusted to baseline values (day 8); and for therapeutic treatment this was from days 14 to day 20, adjusted to baseline values at day 14. ANOVA using Dunnet method was utilized to evaluate differences between each of the study groups and the combined treatment group (CF101+MTX): for prophylactic treatment this was from days 20 to 25, adjusted to baseline values (day 8); and for therapeutic treatment this was from days 14 to 20, adjusted to baseline values at day 14. The data were analyzed using SAS software (SAS Institute, Cary, NC, USA).

The Student's *t*-test was used in the Westen blot analysis, and *P *< 0.05 was considered statistically significant.

## Results

### Effect of MTX, CF101, and MTX+CF101 treatment on development of AIA in rats

**Figure 1:**
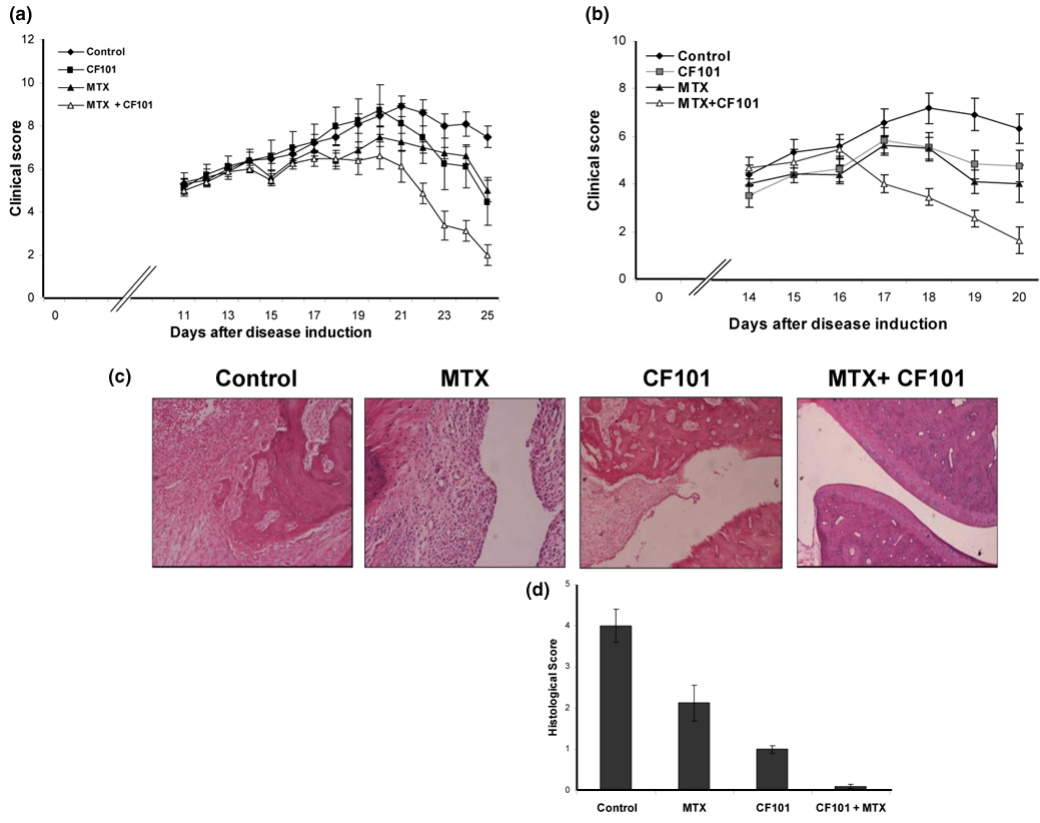
Effect of CF101 and prophylactic or therapeutic MTX treatment on AIA rats. Rats were injected subcutaneously at the tail base with 100 μl of a suspension composed of incomplete Freund's adjuvant and 10 mg/ml heat killed *Mycobacterium tuberculosis*. **(a) **Clinical score. Combined treatment with MTX (prophylactic treatment) + CF101 yield significantly lower values than treatment with each of the agents alone; also, all treatments yielded lower scores than control group. **(b) **The clinical score with combined therapeutic MTX treatment + CF101 was significantly lower than that in the other groups. **(c, d) **Morphopathological score. In AIA animals complete distruction of the cartilage and the bone, as well as severe inflammation in the hind paws, was noted. Treatment with a combination of MTX and CF101 preserved the normal features of the paw. AIA, adjuvant-induced arthritis; MTX, methotrexate.

In the prophylactic treatment study, about 21 days after immunization most of the vehicle-treated animals progressively developed arthritis. Comparing the four groups using ANOVA, CF101 treatment (100 μg/kg, given orally twice daily, starting on onset of disease) and MTX treatment (given once weekly, starting on day 3 after disease induction) resulted in a statistically significant difference between the study groups and the control group (Figure 1a; *P *< 0001). In order to identify the source of those differences, ANOVA with the Dunnet method was used. In general, up to day 20 a differences in trends were observed between the groups, but the differences were not statistically significant. From day 20 on, a clear trend toward lower values in the combined treatment group (MTX+CF101) was observed, as compared with the control group and each of the other treatment groups (Figure 1a).

In the therapeutic treatment study, in which all of the treatments were initiated at the onset of disease, clinical score in the MTX and CF101 combined treatment group was found to be statistically significantly lower than that in the control group. Moreover, clinical score in the group receiving combined treatment with MTX and CF101 was always lower than that the other groups. From day 17 the trend becomes statistically significant (Figure 1b).

Histological evaluation of the paws in the vehicle-treated arthritic animals revealed complete distruction of cartilage and bone, which were replaced by granulation tissue. Severe inflammation infiltration was also noted. In the MTX treated group bone destruction was also observed, with a few remnants of bone spicutels seated in the granulation tissue. The degree of inflammatory infiltration was less than that observed in the control group, resulting in a 47% decrease in histological score. CF101 treatment resulted in a 75% decrease in histological score, which was manifested as a mild inflammatory infiltration. The articular space, cartilage, bone and bone marrow appeared normal. Treatment with MTX in combination with CF101 was associated with normal cartilage and bone architecture, and with an histological score that was almost zero (Figure 1c,d).

### Effect of MTX, CF101 and MTX+ CF101 treatment on exhibition and expression of A_3_AR in paw and PBMCs from AIA rats

**Figure 2:**
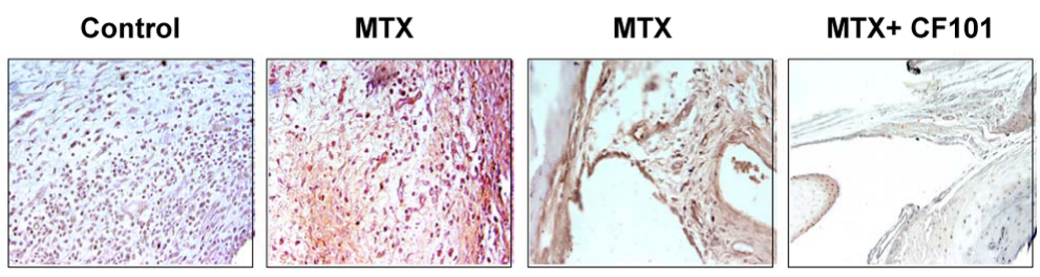
Immunohistochemistry analysis of A_3_AR expression in paws from AIA rats. We conducted immunohistochemistry staining of paraffin-embedded sections of paw from MTX-treated, CF101-treated and MTX+CF101-treated AIA rats. MTX treatment induced receptor expression, and treatment with CF101 alone or in combination with MTX resulted in receptor downregulation. A_3_AR, A_3 _adenosine receptor; AIA, adjuvant-induced arthritis; MTX, methotrexate.

Immunohistochemistry staining of paw sections derived from AIA rats revealed the of A_3_AR exhibition in the control paw, which was markedly increased with MTX treatment. In the CF101-treated group, A_3_AR exhibition was much lower and the receptor was barely detected at all in MTX+CF101-treated paw sections (Figure 2).

**Figure 3:**
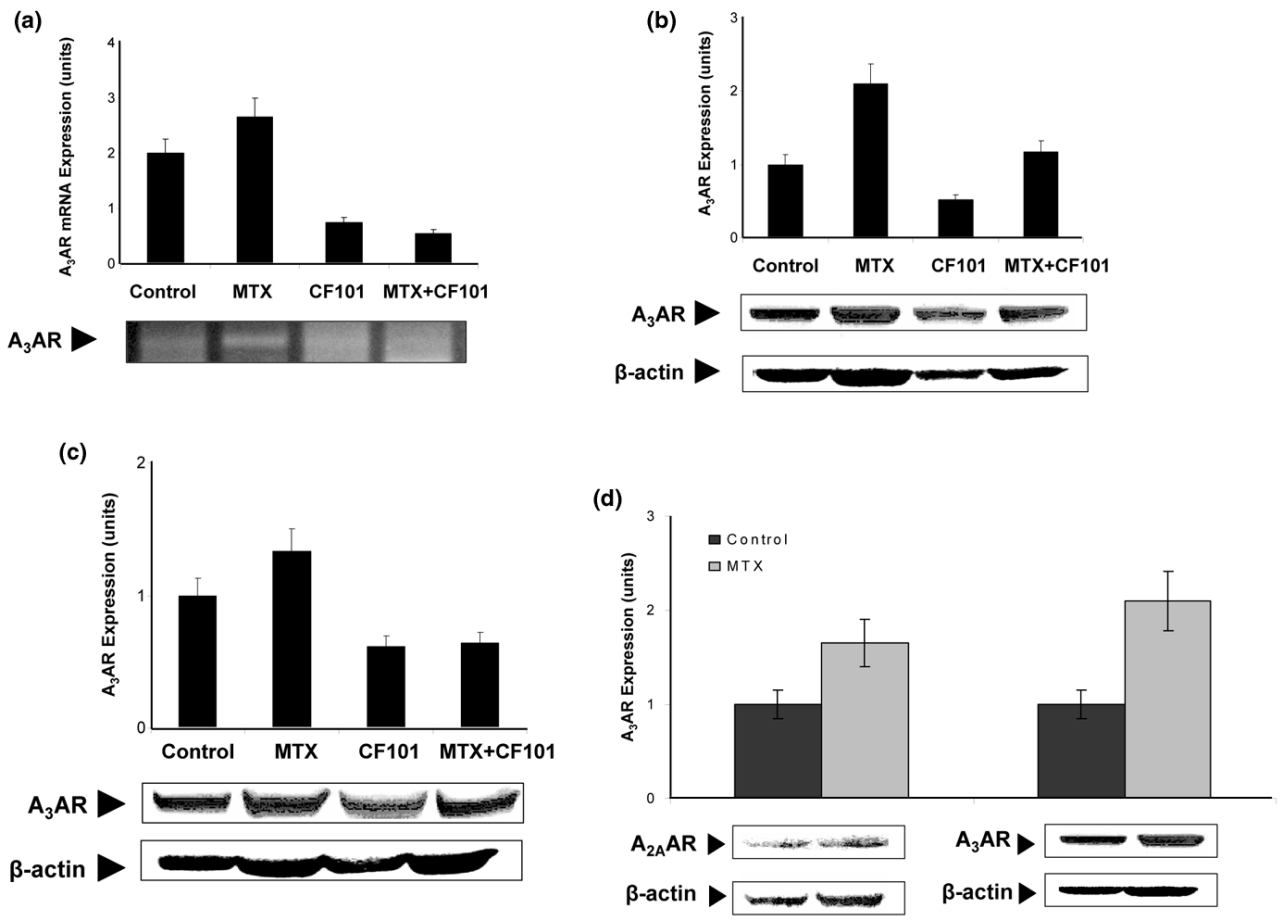
*Ex vivo *analysis of A_3_AR expression level in paw and PBMCs from AIA rats. **(a, b) **MTX treatment induced expression of A_3_AR mRNA and protein, and CF101 treatment resulted in receptor downregulation. **(c) **This was reflected in the PBMCs. **(d) **Expression of A_2A_AR was also induced in paw extracts derived AIA rats treated with MTX. A_3_AR, A_3 _adenosine receptor; AIA, adjuvant-induced arthritis; MTX, methotrexate; PBMC, peripheral blood mononuclear cell.

Analysis of mRNA and protein A_3_AR expression in paw extracts from the MTX-treated group revealed an increase compared with the vehicle-treated group. In the CF101-treatment and the MTX+CF101-treated groups, receptor downregulation was noted, supporting previous studies demonstrating that receptor downregulation represents a functional response to the agonist (Figure 3a,b). In PBMCs from the animals treated with MTX, CF101 and MTX+CF101, similar results to those noted in the paw extracts were recorded (Figure 3c).

We further examined the effect of MTX on expression of A_3_AR protein compared with A_2A_AR protein in paw extracts. Figure 3d shows that the expression levels of both receptors is increased with MTX treatment.

**Figure 4:**
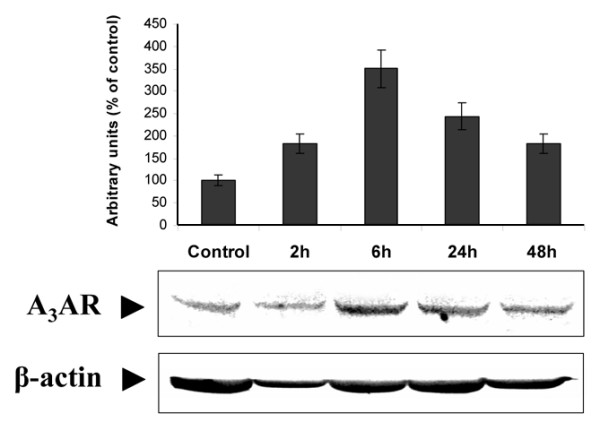
Effect of dipyridamole on the expression of A_3_AR in PBMCs from naïve rats. Dipyridamole (5 mg/kg intraperitoneally) was administered once to naïve rats and blood samples were drawn 2, 6, 24 and 48 hours after dipyridamole administration. Dipyridamole induced A_3_AR upregulation in PBMCs derived from treated rats. A_3_AR, A_3 _adenosine receptor; PBMC, peripheral blood mononuclear cell.

To explore the molecular mechanism involved in the increased expression of A_3_AR with MTX treatment, we assumed this phenomenon to be attributed to elevated adenosine levels in the cell microenvironment. We thus treated naïve rats with dipyridamole, a nucleoside transporter inhibitor that increases extracellular adenosine concentration. Indeed, dipyridamole induced A_3_AR over-expression in PBMCs from treated rats (Figure 4).

### A_3_AR expression in PBMCs from RA patients

**Figure 5:**
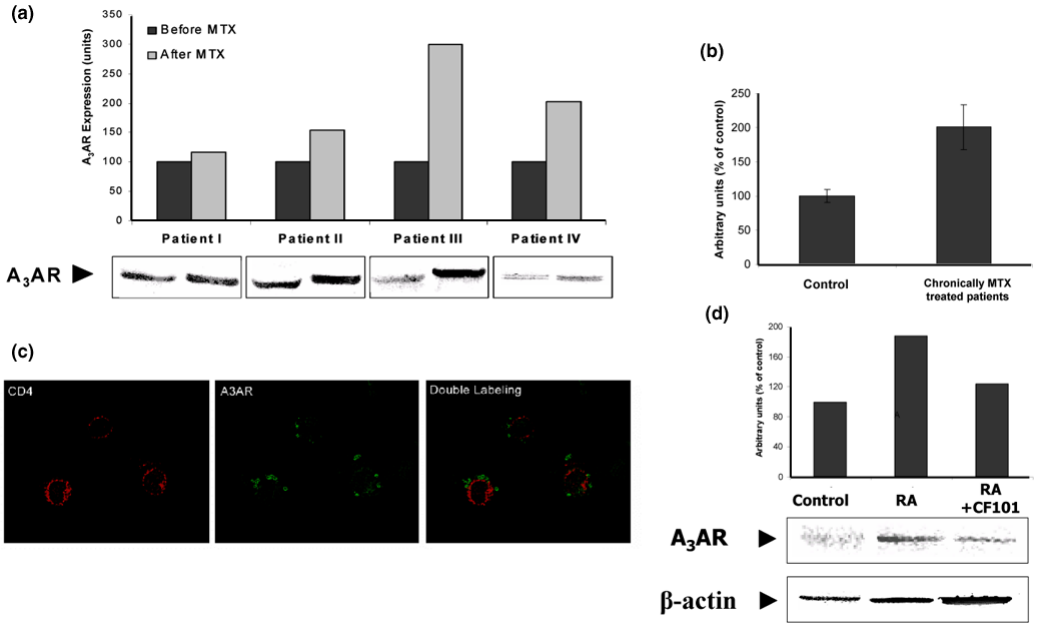
A_3_AR expression level in PBMCs from RA patients. **(a) **Newly MTX treated RA patients. Western blots of the four patients at baseline and 10 weeks after MTX treatment are presented. Representative Western blots are shown at the bottom. **(b) **RA patients undergoing chronic treatment with MTX. Results are expressed as means ± standard error for 30 patients. **(c) **Double-stained immunofluorecence analysis of PBMCs from RA patients. A_3_AR expression on the cell surface of the CD4^+ ^T cells was found. **(d) **Incubation of PBMCs from MTX-treated RA patients for 1 hour in RPMI 1640 supplemented with 10% foetal bovine serum in the presence of with CF101 (10 nmol/l) resulted in decreased expression of A_3_AR. A_3_AR, A_3 _adenosine receptor; AIA, adjuvant-induced arthritis; MTX, methotrexate; PBMC, peripheral blood mononuclear cell; RA, rheumatoid arthritis.

Our next step was to examine A_3_AR expression level in PBMCs from MTX-treated RA patients. Figure 5a shows four blots from newly MTX treated RA patients. Upregulation of A_3_AR expression was observed in all patients after 10 weeks of MTX treatment. There was a marked statistically significant increase (*P *< 0.01) in A_3_AR expression in PBMCs from RA patients undergoing chronic MTX treatment (*n *= 30) in comparison with healthy individuals (Figure 5b). No correlation between A_3_AR expression level, MTX dose, or DAS28 (Disease Activity Score) was found.

In addition, we performed double immunofluorecence staining on PBMCs from RA patients to identify A_3_AR on the cell surface of CD4^+ ^T lymphocytes. Figure 5c shows massive A_3_AR staining on the CD4^+ ^T cells.

In an additional set of experiments we looked at the *in vitro *effect of CF101 on A_3_AR protein level in PBMCs from MTX-treated RA patients. The cells were cultured in the presence and absence of CF101 for 1 hour. Figure 5d shows A_3_AR over-expression in the RA samples compared with control samples. CF101 treatment induced A_3_AR downregulation, reflecting the response of PBMCs to the drug; this suggests that receptor expression may have utility as a biological prediction marker.

### Effect of MTX+CF101 on A_3_AR expression level in PHA-stimulated PBMCs from healthy individuals

**Figure 6:**
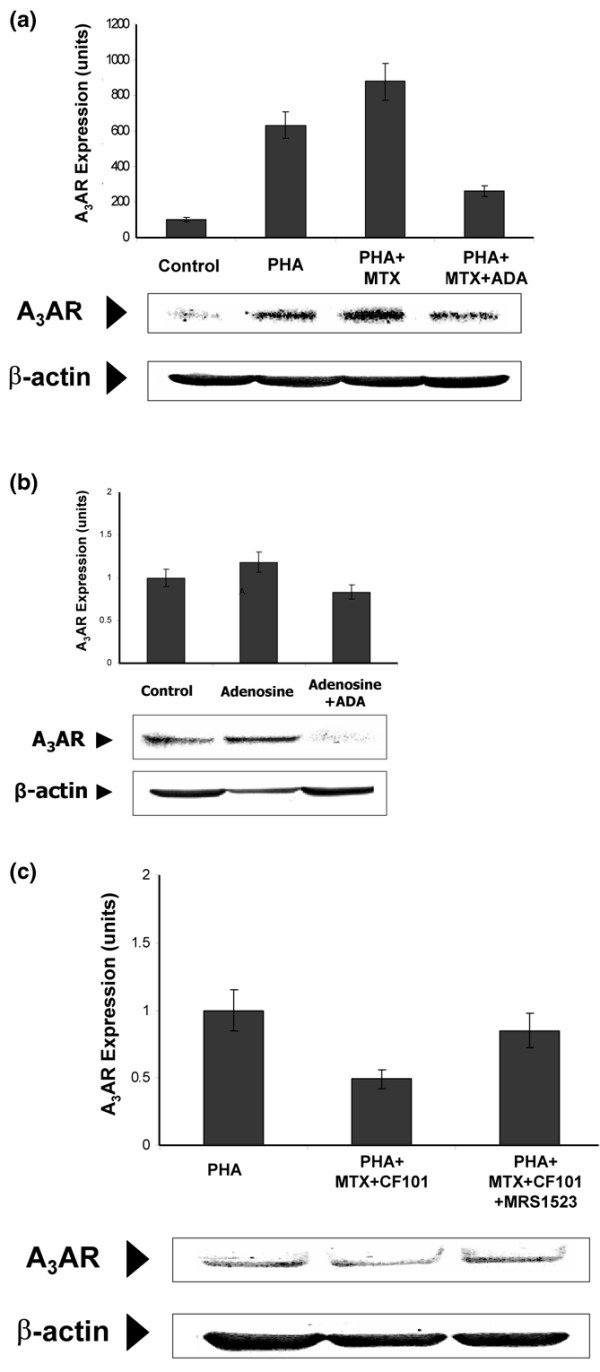
Effect of MTX on A_3_AR expression level on PBMCs from healthy individuals. **(a) **PBMCs (2 × 10^6 ^cells/ml) from healthy individuals were incubated in RPMI 1640 supplemented with 10% foetal bovine serum and activated with PHA (5 μg/ml) for 24 hours. MTX (1 μmol/l) was added for an additional 27 hours and ADA (1 unit/ml) for the last 3 hours. A_3_AR expression level was induced by MTX treatment. The introduction of ADA to the culture system reverted this effect and induced downregulation of the receptor level. **(b) **PBMCs (2 × 10^6 ^cells/ml) were incubated for 27 hours with adenosine (25 μmol/l) and ADA (1 unit/ml) was added to the culture system for the last 3 hours. Adenosine induced upregulation of A_3_AR expression level whereas the addition of ADA decreased the receptor level. **(c) **PBMCs from healthy individuals were incubated with 10% foetal bovine serum and activated with PHA (5 μg/ml) for 24 hours. MTX (1 μmol/l) was added for an additional 27 hours and CF101 (10 nmol/l), in the presence or absence of MRS1523 (10 nmol/l), was introduced into the culture system for the last 3 hours. CF101 introduction to MTX-treated, PHA-activated PBMCs induced receptor downregulation, and the MRS1523 counteracted this effect. A_3_AR, A_3 _adenosine receptor; ADA, adenosine deaminase; MTX, methotrexate; PBMC, peripheral blood mononuclear cell; PHA, phytohemagglutinin; RA, rheumatoid arthritis.

To further investigate the effect of MTX on A_3_AR expression, we used an *in vitro *system of PHA-stimulated PBMCs from healthy individuals. We found that A_3_AR expression level was increased on MTX treatment. Also, introduction of ADA to the culture system reverted this effect, suggesting that receptor over-expression was induced by adenosine, a metabolite of MTX (Figure 6a). Finally, the natural ligand adenosine induced an increase in A_3_AR expression when added to the culture system, supporting the notion presented above that adenosine acts as mediator of the MTX effect (Figure 6b).

Introduction of CF101 to MTX-treated, PHA-stimulated cells induced receptor downregulation, which was counteracted by the A_3_AR antagonist MRS1535 (Figure 6c).

## Discussion

The present study shows that combined treatment with CF101 and MTX has an additive anti-inflammatory effect, which is indicated by a decrease in the clinical and pathological manifestations of AIA. Mechanistically, MTX induced an increase in A_3_AR expression level in inflamed tissues and in PBMCs, thereby rendering the cells more susceptible to CF101 treatment. Interestingly, MTX given either in prophylactic or therapeutic mode in combination with CF101 resulted in the same additive effect.

To explore the mechanism underlying the efficacy of the combined treatment, we first examined the mode of action of MTX. It acts as an antagonist of folic acid, subsequently inhibiting the synthesis of purines and pyrimidines. It was further suggested that the anti-inflammatory effect of MTX is due to adenosine, which is known to exert potent anti-inflammatory effect [3,4]. More specifically, MTX polyglutamates inhibit the activity of the enzyme aminoimidazolecarbox-amidoadenosineribonucleotide transformylase [17,18]. This enzyme has a direct inhibitory effect on two additional enzymes: ADA, which metabolizes adenosine to inosine; and AMP deaminase, which converts adenosine to AMP. This chain of events results in intracellular accumulation of adenosine, which is then released into the extracellular environment [19]. The working hypothesis of the present study was that the increase in adenosine level may act via an autocrine pathway and induce the expression of its own receptors, in this case A_3_AR. Indeed, MTX treatment increased both mRNA and protein levels of A_3_AR in the inflammatory tissue (paw) of AIA rats, indicating that increased gene expression and translation took place. An increase in protein A_3_AR expression was also noted in the PBMCs from the animals. Interestingly, increased A_2A_AR protein levels were also noted in paw samples from MTX-treated animals. Ravid and coworkers [20] have shown that activation of A_2A_R increases A_3_AR promoter activity. It could therefore be suggested that over-expression of A_2A_AR plays a role in the increased level of A_3_AR that occurs with MTX treatment. Moreover, A_3_AR protein level was raised in RA patients treated with MTX. This was attributed to the increase in A_3_AR expression detected on CD4^+ ^T cells from RA patients compared with CD4^+ ^T cells from healthy individuals.

In this report we present two lines of experimental evidence that support a role for adenosine in modulating A_3_AR expression in response to MTX treatment. *In vitro *studies with PBMCs showed that MTX treatment induced an increase in A_3_AR expression that was reversed on treatment with ADA. Adenosine mimicked the effect of MTX and induced an elevation in A_3_AR expression. *In vivo *dipyridamole, an inhibitor of nucleoside transporters, known to increase adenosine levels, induced A_3_AR over-expression in PBMCs from treated rats. These data led to the conclusion that MTX, via the metabolite adenosine, induces elevation in A_3_AR expression.

Receptor density was previously reported as an important factor that controls cell response to a given agonist [21-23]. It may be suggested that A_3_AR upregulation, mediated by MTX, preconditions cells to the effect of CF101, resulting in a more potent anti-inflammatory effect.

An additional important finding of the study is that inflammatory cells and PBMCs from CF101-treated AIA rats, as well as the PBMCs cultured *in vitro *with CF101, responded to agonist treatment and to combined treatment with receptor downregulation. The A_3_AR antagonist MRS 1353 counteracted the downregulation induced by the combined treatment. This finding supports the notion presented above that the effect of CF101 alone or in combination with MTX is solely mediated via A_3_AR, without the involvement of additional receptors. Our earlier studies in tumor cells showed that, on binding of an agonist such as CF101 to tumor cells, receptor is internalized and degraded within the cytoplasm [24,25]. This is followed by initiation of downstream signal transduction pathways, leading to inhibition of tumor growth. A few hours later receptor is resynthesized and recycled to the cell surface. Recently, we reported a similar chain of events in the synovial cells of AIA rats treated with CF101. A_3_AR receptor levels were downregulated on agonist treatment, followed by modulation of cell signaling proteins that belong to the NF-κB signal transduction pathway, resulting in decreased expression of TNF-α and inhibition of the inflammatory process [12]. It thus seems that if A_3_AR upregulation is characteristic of inflammation, then CF101 treatment induces receptor downregulation, which reflects receptor functionality, and modulation of key signaling proteins that control the inflammatory process. Interestingly, introduction of CF101 to *in vitro *culture of PBMCs derived from MTX-treated RA patients resulted in A_3_AR downregulation. The ability to assess *in vitro *the response to CF101 may be utilized in the future to predict the response of individual patients to the drug before treatment.

The mechanism presented in this study by which MTX elevates A_3_AR expression may also account for the anti-inflammatory effect of this drug when given as a standalone therapy. Under physiological conditions A_3_AR is not activated because adenosine has the lowest affinity value to this receptor (A_2A _> A_1 _> A_2B _> A_3_). On MTX treatment the adenosine level goes up, thereby activating A_3_AR and resulting in an anti-inflammatory effect [3,4,26].

High expression levels of A_3_AR in inflamed or activated cells were previously reported in other inflammatory conditions. A selective, approximately 10-fold upregulation of A_3_AR mRNA and protein was consistently found in nonpigmented ciliary epithelium of eyes of patients with pseudo-exfoliation syndrome, with and without glaucoma, as compared with normal and glaucomatous control eyes [27]. Blair and coworkers [28] showed that A_3_AR transcript abundance is greater in lung tissue and eosinophils from individuals with airway inflammation than in normal lung. A_3_AR elevation was also reflected in eosinophils derived from the peripheral blood of the same patients. Treatment of eosinophils with IB-MECA inhibited platelet-activating factor induced eosinophil chemotaxis [28]. Moreover, Gessi and coworkers [29] reported that A_3_AR is induced in activated PBMCs from healthy individuals, and further demonstrated that the CD4^+ ^T cells are the subpopulation of cells that over-express the receptor. Taken together, it may be concluded that A_3_AR upregulation is a characteristic of activated cells, and is noted in cells of inflammatory origin and is reflected in PBMCs.

To summarize, enhanced anti-inflammatory effect takes place on treatment of AIA rats with the combination of MTX and CF101. This effect is mediated via adenosine, which accumulates in cells on MTX treatment, leading to increased levels of A_3_AR, thereby rendering the cells more sensitive to the effect of CF101. We recently reported a positive response of RA patients to CF101 treatment (as a standalone therapy) in a phase IIa study [15]. The results of the present study provide justification for a phase IIb study combining CF101 with MTX treatment in a population of RA patients.

## Conclusion

Using an *in vivo *model of AIA in rats, we demonstrated that combined treatment with CF101 and MTX resulted in an additive anti-inflammatory effect. Mechanistically, we found that MTX induced an increase in the expression level of A_3_AR, rendering the inflammatory cells more susceptible to CF101 treatment. Adding further support to these findings were data from the MTX-treated RA patients, who exhibited A_3_AR over-expression in PBMCs.

## Abbreviations

A_3_AR = A_3 _adenosine receptor; ADA = adenosine deaminase; AIA = adjuvant-induced arthritis; BSA = bovine serum albumin; IB-MECA = 1-deoxy-1-[6-[[(3-iodophenyl)methyl]amino]-9H-9-yl]-*N*-methyl-β-d-ribofura-nuronamide; MTX = methotrexate; NF-κB = nuclear factor-κB; PBMC = peripheral blood mononuclear cell; PBS = phosphate-buffered saline; PHA = phytohemagglutinin; RA = rheumatoid arthritis; RT-PCR = reverse transcription polymerase chain reaction; TNF = tumour necrosis factor.

## Competing interests

The authors declare that they have no competing interests.

## Authors' contributions

OA, CH, ML and BF conducted the molecular biology work. ZA conducted the *in vivo *studies. FM conducted data management for the patients. FFS conducted patient sample handling and patient assessment. RT, RA, AH, LY, MY, MR, TM and LP collected blood samples and patient data. SBY precipitate in study design and data analysis, and helped to draft the manuscript. FP conceived the study, participated in the study design and data analysis, and drafted the manuscript.

## Figures and Tables

**Table 1:** Characteristic of patients newly and chronically treated with MTX

Characteristic	RA patients	
	
	Newly MTX treated	Chronically MTX treated
Age (years)	48.5 ± 5.89	59.58 ± 2.42
Disease duration (years)	4.37 ± 2.07	6.6 ± 0.97
CRP (mg/l)	10.75 ± 2.01	9.04 ± 1.65
ESR (mm/hour)	31.75 ± 0.63	33.3 ± 4.39
Swollen joint count	13.25 ± 2.56	3.19 ± 0.88
Tender joint count	10.5 ± 2.63	5 ± 0.86
Patient global assessment (VAS)		32.26 ± 4.41
CRP (DAS28-4)		3.23 ± 0.31
ESR (DAS28-4)		4.11 ± 0.3

Values are expressed as mean ± standard error. CRP, C-reactive protein; DAS, Disease Activity Score; ESR, erythrocyte sedimentation rate; MTX, methotrexate; RA, rheumatoid arthritis; VAS, visual analogue scale.
